# Expression of matrix metalloproteinases and their inhibitors in different immunohistochemical-based molecular subtypes of breast cancer

**DOI:** 10.1186/1471-2407-14-959

**Published:** 2014-12-16

**Authors:** Ga-Eon Kim, Ji Shin Lee, Yoo-Duk Choi, Kyung-Hwa Lee, Jae Hyuk Lee, Jong Hee Nam, Chan Choi, Sung Sun Kim, Min Ho Park, Jung Han Yoon, Sun-Seog Kweon

**Affiliations:** Deparment of Pathology, Chonnam National University Medical School, Gwangju, Republic of Korea; Department of Surgery, Chonnam National University Medical School, Gwangju, Republic of Korea; Department of Preventive Medicine, Chonnam National University Medical School, Gwangju, Republic of Korea

**Keywords:** Breast cancer, Molecular subtype, Immunohistochemistry, Matrix metalloproteinase, Tissue inhibitor of metalloproteinase

## Abstract

**Background:**

Metalloproteinases (MMPs) and their tissue inhibitors of metalloproteinases (TIMPs) are involved in several key pathways of tumor growth, invasion and metastasis, but little is known about their expression according to different molecular subtypes of breast cancer. The aims of this study were to assess the prevalence and clinical significance of MMP and TIMP expression in invasive breast cancer and to determine its association with immunohistochemical-based molecular classification.

**Methods:**

Tissue microarray sections were immunostained for estrogen receptor-α (ER-α), progesterone receptor (PR), human epidermal growth factor receptor 2 (HER2), cytokeratin (CK) 5/6, epidermal growth factor receptor (EGFR) and with specific antibodies against MMP-1, 2, 7, 9, 11, 13, and 14 and TIMP-1, 2, and 3. Based on the immunostaining data from five of the markers used (ER-α, PR, HER2, EGFR and CK5/6), three major subtypes (123 luminal A, 31 basal-like, and 17 HER2-overexpressing) were selected.

**Results:**

Statistically significant differences in the expression of MMPs and TIMPs among the three subtypes were found in tumoral MMP7 (*P* = 0.005), tumoral MMP-9 (*P* = 0.000), tumoral MMP-13 (*P* = 0.016) and stromal MMP-13 (*P* = 0.016). The incidence of tumoral MMP-9 expression in the HER2-overexpressing subtype was significantly higher than in the luminal A subtype (*P* = 0.021). Tumoral MMP-9 and stromal MMP-13 expression were significantly higher in the HER2-overexpressing subtype than in the basal-like subtype (*P* = 0.000 and *P* = 0.016, respectively). Tumoral MMP-7 expression was significantly higher in the basal-like subtype compared to luminal A (*P* = 0.007) and HER2-overexpressing subtype (*P* = 0.004). Tumoral MMP-13 showed a higher expression in the basal-like subtype than in the HER2-overexpressing subtype (*P* = 0.010). In multivariate analysis, stage and stromal MMP-1 expression were significantly related to overall survival. Stage was of independent prognostic significance for disease-free survival.

**Conclusion:**

We found some variations in MMP and TIMP expression among the immunohistochemical-based molecular subtypes of breast carcinomas, suggesting differences in their tumor pathophysiology. Additional studies are needed to determine the mechanisms underlying the differences of MMP and TIMP expression in the molecular subtypes for the development of specific therapeutic targets for breast cancer subtypes.

## Background

Breast cancer is the second most common malignancy in Korean women representing 16% of all female cancers [1]. Breast carcinoma encompasses a group of very heterogeneous diseases including a number of distinct entities with specific pathological features and biological behavior [2, 3].

Microarray profiling of breast carcinoma has identified five distinct subtypes of tumors (luminal A, luminal B, normal breast-like, human epidermal growth factor receptor 2 (HER2)-overexpressing, and basal-like) that are associated with different clinical outcomes [4–7]. Although this classification system is based on extensive genetic profiling assays, a simplified method of classification based on immunohistochemical surrogates is appealing and more clinically useful. Based on the immunostaining data from five markers [estrogen receptor-α (ER-α), progesterone receptor (PR), HER2, cytokeratin (CK) 5/6, and epidermal growth factor receptor (EGFR)], breast carcinoma can be categorized as luminal A (ER-α + and/or PR+ and HER2-); luminal B (ER-α + and/or PR+ and HER2+); HER2-overexpressing (ER-α- and PR- and HER2+); basal-like (ER-α-, PR-, HER2- and EGFR or CK 5/6+); and unclassified (ER-α-, PR-, HER2-, EGFR-, and CK 5/6-) [8, 9]. Compared with the luminal subtype, basal-like and HER2-overexpressing breast cancers are associated with worse overall and disease-free survival rates [6, 7]. Basal-like carcinoma has a triple–negative phenotype (ER-α-, PR-, and HER2-); as a result, the majority of these tumors cannot be managed effectively with existing targeted treatment (including Trastuzumab and hormonal treatments) [10]. Therefore, there is a need for the development of new therapies specifically for basal-like breast cancer.

Matrix metalloproteinases (MMPs) and their tissue inhibitors of metalloproteinases (TIMPs) act in concert to control extracellular matrix turnover [11, 12]. MMP and TIMP expression is altered in both benign and malignant tumors, as well as in invasion and metastasis which require breakdown and removal of the extracellular matrix [13, 14]. The central role of MMPs and TIMPs in tumor invasion and metastasis makes them an attractive target for drug development [15].

Previous studies have shown the expression and activity of MMPs to be linked to the advanced stage of breast cancer, increased invasion of tumor cells and building of metastatic formations [16–18]. Likewise, it has been reported that TIMPs may be overexpressed and/or related to clinical outcome of breast carcinoma [16]. However, the association between MMP and TIMP expression and the distinct molecular subtypes of breast carcinoma has not been well investigated [17, 19].

We designed this study to analyze different expression levels of MMPs and TIMPs in breast carcinoma with respect to immunohistochemical-based molecular classification and to determine their relationship to other clinical-pathological factors. MMPs and TIMPs, which are known to be involved in breast carcinogenesis (MMP-1, 2, 7, 9, 11, 13, and 14 and TIMP-1, 2, and 3), were selected and assessed using the immunohistochemistry of three major subtypes of invasive breast carcinomas (luminal A, basal-like, and HER2-overexpressing); based on the immunohistochemical findings of ER-α, PR, HER2, EGFR, and CK5/6.

## Methods

### Case selection

Histologic files of Chonnam National University Hospital, Gwangju, Korea from the period between 1997 and 2002 were searched for invasive breast carcinoma. We selected 204 cases with a minimum of 10 years of follow-up. Tumor tissue was obtained from patients with unilateral breast carcinoma after surgical resection. We excluded patients with distant metastases at the time of initial diagnosis or with bilateral breast carcinoma at diagnosis. Furthermore, patients who had received neoadjuvant therapy, or who had a prior history of any kind of cancer, were excluded from this study. All samples were obtained with informed consent under protocols approved by the institutional review board of the Chonnam National University Hospital. Full clinical and pathological data were collected and known for all participants.

### Tissue microarray construction

The arrays were constructed with a 1.5 mm punch on the Beecher arrayer. The array layout in the grid format was designed using Microsoft Excel. Hematoxylin and eosin-stained sections were reviewed and the area of interest was marked out on the slide. Using a marker pen, the corresponding region was circled on the archival ‘donor’ paraffin block. The samples were then arrayed on to a ‘recipient’ blank block. Each sample was arrayed in triplicate to minimize tissue loss and overcome tumor heterogeneity.

### Immunohistochemistry and silver-enhanced *in situ*hybridization

Tissue microarray sections were immunostained for ER-α, PR, HER2, CK 5/6, and EGFR and specific antibodies against MMP-1, 2, 7, 9, 11, 13, and 14 and TIMP-1, 2, and 3. Automated immunohistochemical staining was performed using the Bond-max system (Leica Microsystems, Bannockburn, IL), which is a device able to process up to 30 slides at a time. Slides carrying tissue sections that were cut from paraffin-embedded tissue microarray blocks were labeled and dried for 1 hour at 60°C. These slides were then covered by Bond Universal Covertiles (Leica Microsystems) and placed into the Bond-max instrument. All subsequent steps were performed by the automated instrument according to the manufacturer’s instructions (Leica Microsystems), in the following order: (1) deparaffinization of tissue on the slides using Bond Dewax Solution (Leica Microsystems) at 72°C for 30 minutes; (2) heat-induced epitope retrieval (antigen unmarking) with Bond Epitope Retrival Solution 1 (Leica Microsystems) for 20 minutes at 100°C; (3) peroxide block placement on the slides for 5 minutes at ambient temperature; and (4) incubation with ER-α (1:35, clone 1D5, DakoCytomation, Glostrup, Denmark), PR (1:50, clone PgR 636, DakoCytomation), HER2 (1:250, DakoCytomation), CK5/6 (1:50, clone D5/6 B4, DakoCytomation), EGFR (1:200, clone H11, DakoCytomation), MMP-1 (1:50, Thermo Fisher Scientific, Fremont, CA), MMP-2 (1:25, clone A-Gel VC2, Thermo Fisher Scientific), MMP-7 (1:200, clone ID2, Thermo Fisher Scientific), MMP-9 (1:50, Thermo Fisher Scientific), MMP-11 (1:100, clone SL3.05, Thermo Fisher Scientific), MMP-13 (1:25, clone VIIIA2, Thermo Fisher Scientific), MMP-14 (1:50, Thermo Fisher Scientific), TIMP-1 (1:25, clone 102D1, Thermo Fisher Scientific), TIMP-2 (1:200, clone 3A4, Thermo Fisher Scientific), and TIMP-3 (1:50, clone Z188, Santa Cruz Biotechnology, Santa Cruz, CA) primary antibodies for 15 minutes at ambient temperature; (5) incubation with Post Primary Regent (Leica Microsystems) for 8 minutes at ambient temperature, followed by washing with Bond Wash Solution (Leica Microsystems) for 6 minutes; (6) Bond Polymer (Leica Microsystems) placement on the slides for 8 minutes at ambient temperature, followed by washing with Bond Wash and distilled water for 4 minutes; (7) color development with DAB (3,3’-diaminobenzidine tetrahydrochloride) as chromogen for 10 minutes at ambient temperature; and (8) hematoxilyn counterstaining for 5 minutes at ambient temperature, followed by mounting of the slides. Paraffin sections of normal breast were used as positive controls for CK5/6, sections of breast carcinoma were used for ER-α, PR, HER2, MMPs, and TIMPs positive controls, and a squamous cell carcinoma of the skin was used for EGFR’s positive control. The primary antibody incubation step was omitted in the negative control.

Tissue microarrays were digitized (Aperio Technologies, Vista, CA) and semi-quantified estimation for immunoreactivity was performed in all cases. Tumor cells that showed nuclear staining for ER-α or PR were considered ER-α + or PR+, whereas all ER- or PR- cases showed a complete absence of tumor cell staining. Of note, low positive ER or PR (1-10% of tumor cell nuclei staining) and positive ER or PR (>10% of tumor cell nuclei staining) were collapsed into a single ER or PR ‘positive’ category for the purposes of this analysis. Results of HER2 immunostaining were scored according to the ASCO/CAP guidelines. HER2 immunostaining was considered positive when strong (3+) membranous staining was observed in at least 30% of tumor cells, whereas cases with 0 to 1+ were regarded as negative. Cases with equivocal (2+) result for HER2 immunostaining were retested by silver-enhanced *in situ* hybridization (SISH). HER2 SISH testing and scoring were performed as described previously below [20]. Briefly, INFORM® HER2 DNA and Chromosome 17 probes (Ventana Medical System) were done on the microarray sections using the Benchmark® automatic immunostaining device in accordance with the Ventana’s protocol. HER2 signals were scored according to the 2008 ASCO/CAP guidelines. Any degree of cytoplasmic immunostaining for CK 5/6 and any degree of distinct membranous staining for EGFR were considered as positive expression. A case was classified as positive if there was positive staining in any of the three cores from that case and negative if there was no immunostaining.

MMP and TIMP immunoreactivity in the tumor tissue and in the surrounding stromal tissue was evaluated. We could differentiate tumor cells from stromal cells based on their distinctive morphologies. Tumor cells are larger than stromal cells. In addition, tumor cells show nucleoli and are arranged in tubules, irregular nests, or solid sheets. Stromal cells are fibroblasts or mononuclear inflammatory cells. A scoring system was used to describe both the intensity of staining (negative, weak, moderate, and strong) and the proportion of positive cells (0%, 1-25%, 26-50%, 51-75%, and 76-100%) in each case. To enable the analysis of the individual immunostaining results, integer values were assigned to the intensity score (0–3) and to the proportion of stained cells (0–4). The percentages of MMP and TIMP immunoreactive cells were evaluated from two separate protein stained fields per core under 400x magnification. These values were added together to provide a single integrated score for each MMP or TIMP, and the average data of three cores were used for further analysis. Tumors having a final staining score of >2 were considered positive [5].

### Immunohistochemical-based molecular classification

Cancers were categorized as luminal A (ER-α + and/or PR+ and HER2-); luminal B (ER-α + and/or PR+ and HER2+); HER2-overexpressing (ER-α-, PR-, and HER2+); basal-like (ER-α-, PR-, HER2- and EGFR+ or CK5/6); and unclassified (ER-α-, PR-, HER2-, EGFR-, and CK 5/6-).

### Statistical analysis

Tumor characteristics and expression of MMPs and TIMPs were compared across different breast cancer subtypes using the exact χ^2^ test for categorical data and the nonparametric Kruskal-Wallis test for continuous data. Survival curves were estimated using the Kaplan-Meier method. The distribution of survival was compared using the log-rank test. Multivariate analysis was performed using the Cox’s proportional hazard model. In the multivariate analysis, we included only parameters that achieved statistical significance for relapse-free survival or overall survival in the log-rank test.

For all statistical analyses, the SPSS system for personal computer (version 18.0 for windows; SPSS INC., Chicago, IL) was used and *P* < 0.05 was regarded as statistically significant.

## Results

### Clinicopathologic characteristics

**Figure 1:**
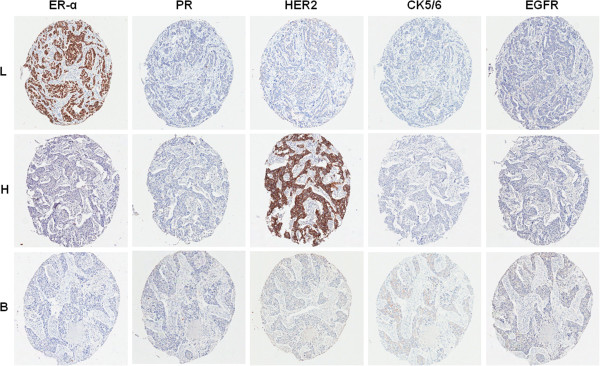
**Representative cases of luminal A (L), HER2-overexpressing (H), basal-like (B) subtype with immunostaining of estrogen receptor-α (ER- α), progesterone receptor (PR), human epidermal growth factor receptor 2 (HER2), cytokeratin (CK) 5/6, and epidermal growth factor receptor (EGFR)**.

Out of the 204 patients with invasive breast carcinoma we surveyd, 123 (60.3%) were luminal A; 17 (8.3%), luminal B; 31 (15.2%), basal-like; 17 (8.3%), HER2-overexpreessing; and 16 (7.8%), unclassified (Figure 1). For the present study, we selected the three most frequent types: luminal A, basal-like, and HER2-overexpressing subtypes. Table 1 shows the clinicopathologic characteristics of the study subjects across the three subtypes of breast cancer. Our results did not show significant differences between the three types with regard to age, tumor size, nodal status, and stage. However, significant differences among the three subtypes of breast carcinoma were found for histologic grade (*P* = 0.000). Both the basal-like and HER2-overexpressing subtypes were associated with a higher grade than the luminal A group (*P* = 0.000 and *P* = 0.000, respectively).

**Table 1:** **Clinicopathologic characteristics of each subtype**

	Luminal A (n = 123)	Basal-like (n = 31)	HER2 (n = 17)	***P***value ^*^
Age (yrs)				0.509
Mean ± SD	46.0 ± 10.5	48.2 ± 10.8	47.5 ± 10.2	
Median (range)	45 (21–89)	46 (30–70)	45 (30–70)	
Grade				0.000
1	25 (20.3%)	0 (0)	0 (0)	
2	72 (58.5%)	3 (9.7%)	8 (47.1%)	
3	26 (21.1%)	28 (90.3%)	9 (52.9%)	
Tumor size (cm)				0.113
2≤	34 (27.6%)	6 (19.4%)	5 (29.4%)	
2-5	77 (62.6%)	17 (54.8%)	8 (47.1%)	
〉5	12 (9.8%)	8 (25.8%)	4 (23.5%)	
Lymph node involvement				0.991
0	63 (51.2%)	16 (51.6%)	9 (52.9%)	
1-3	32 (26.0%)	9 (29.0%)	4 (23.5%)	
4-9	17 (13.8%)	3 (9.7%)	3 (17.6%)	
10	11 (8.9%)	3 (9.7%)	1 (5.9%)	
Stage				0.981
I	27 (22.0%)	6 (19.4%)	4 (23.5%)	
II	65 (52.8%)	17 (54.8%)	8 (47.1%)	
III	31 (25.2%)	8 (25.8%)	5 (29.4%)	

* *P* value obtained using the nonparametric Kruskal-Wallis test for continuous data and the exact chi-square test for categorical data.

### Expression of MMPs and TIMPs

**Figure 2:**
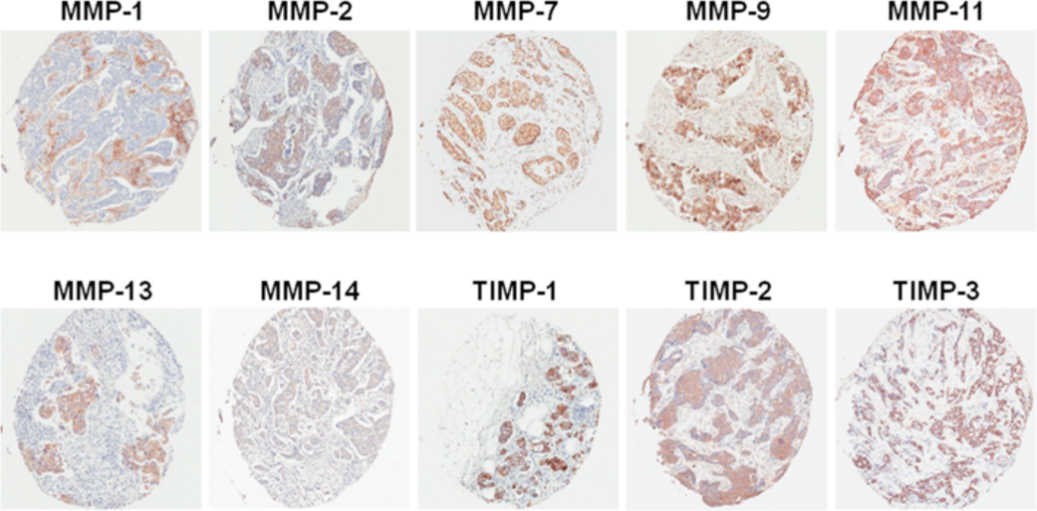
**Examples of tissue microarrays with immunostaining for metalloproteinases (MMPs) and their tissue inhibitors of metalloproteinases (TIMPs).**

Immunostaining data was available for all markers in the basal-like and HER2-overexpressing subtypes. In the luminal A subtype, immunostaining data was available for all markers in 123 cases except for TIMP1. TIMP1 was available for interpretation in 122 of the 123 cases. Figure 2 shows the examples of tissue microarrays with immunostaining for MMPs and TIMPs. Immunostaining for each protein was localized to neoplastic cells but also visible in stromal cells around the tumor. Tumor cells showed a greater expression of MMPs and TIMPs than stromal cells except for MMP-1.

Tumoral MMP-7 (*P* = 0.005), tumoral MMP-9 (*P* = 0.000), tumoral MMP-13 (*P* = 0.016) and stromal MMP-13 (*P* = 0.016) expression showed statistically significant differences among the three subtypes (Table 2). The incidence of tumoral MMP-9 expression in the HER2-overexpressing subtype was significantly higher than in the luminal A subtype (*P* = 0.021) and the basal-like subtype (*P* = 0.000). Stromal MMP-13 expression was significantly higher in the HER2-overexpressing subtype than in the basal-like subtype (*P* = 0.016).

**Table 2:** **Immunohistochemical results of MMPs and TIMPs in each subtype**

Characteristics	Tumor subtype			***P***value ^*^
	Luminal A (n = 123)	Basal-like (n = 31)	HER2 (n = 17)	
	Posistive No/Cases (%)	Positive No/Cases (%)	Positive No/Cases (%)	
MMP 1				
Tumoral	13/123 (10.6)	3/31 (9.7)	2/17 (11.8)	1.000
Stromal	96/123 (78.0)	22/31 (71.0)	13/17 (76.5)	0.470
MMP 2				
Tumoral	47/123 (38.2)	11/31 (35.5)	7/17 (41.2)	0.935
Stromal	4/123 (3.3)	2/31 (6.5)	0/17 (0)	0.641
MMP 7				
Tumoral	100/123 (81.3)	31/31 (100)	12/17 (70.6)	0.005
Stromal	35/123 (28.5)	11/31 (35.5)	5/17 (29.4)	0.767
MMP 9				
Tumoral	28/123 (22.7)	11/31 (35.5)	12/17 (70.6)	0.000
Stromal	6/123 (4.9)	0/31 (0)	0/17 (0)	0.507
MMP 11				
Tumoral	115/123 (93.5)	30/31 (96.8)	17/17 (100)	1.000
Stromal	101/123 (82.1)	29/31 (93.5)	13/17 (76.5)	0.253
MMP 13				
Tumoral	45/123 (36.6)	17/31 (54.8)	12/17 (70.6)	0.016
Stromal	9/123 (7.32)	5/31 (16.1)	5/17 (29.4)	0.016
MMP 14				
Tumoral	67/123 (54.5)	21/31 (67.7)	12/17 (70.6)	0.331
Stromal	42/123 (34.1)	5/31 (16.1)	5/17 (29.4)	0.118
TIMP 1				
Tumoral	64/122 (52.5)	12/31 (38.7)	10/17 (58.8)	0.274
Stromal	22/122 (18.0)	4/31 (12.9)	2/17 (11.8)	0.748
TIMP 2				
Tumoral	62/123 (50.4)	16/31 (51.6)	11/17 (64.7)	0.657
Stromal	16/123 (13.0)	6/31 (19.4)	4/17 (23.5)	0.368
TIMP 3				
Tumoral	100/123 (81.3)	27/31 (87.1)	16/17 (94.1)	0.605
Stromal	45/123 (36.6)	18/31 (58.1)	8/17 (47.1)	0.107

^*^ *P* value obtained using the exact chi-square test.

Tumoral MMP-7 expression was significantly higher in the basal-like subtype compared to the luminal A subtype (*P* = 0.007) and the HER2-overexpressing subtype (*P* = 0.004). Tumoral MMP-13 expression showed a higher expression in the basal-like subtype than in the HER2-overexpressing subtype (*P* = 0.010).

### Correlation with patient survival

Survival data of the three subtypes was available for all 171 patients (mean follow-up 117.3 months, median, 131 months; range, 1–190 months). Fourty-seven patients experienced local recurrence or metastasis (11 with local recurrence and 36 with distant metastases), and 124 remained disease free. There were 50 deaths due to breast carcinoma.

**Figure 3:**
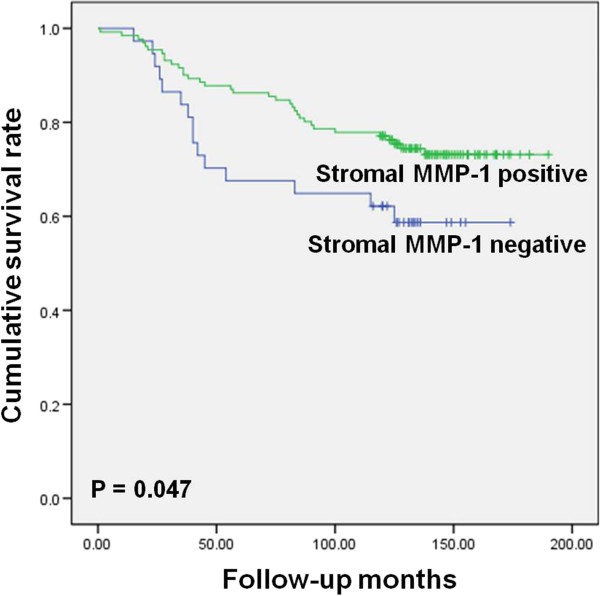
**Kaplan-Meier overall survival curves for stromal MMP-1 expression.**

**Figure 4:**
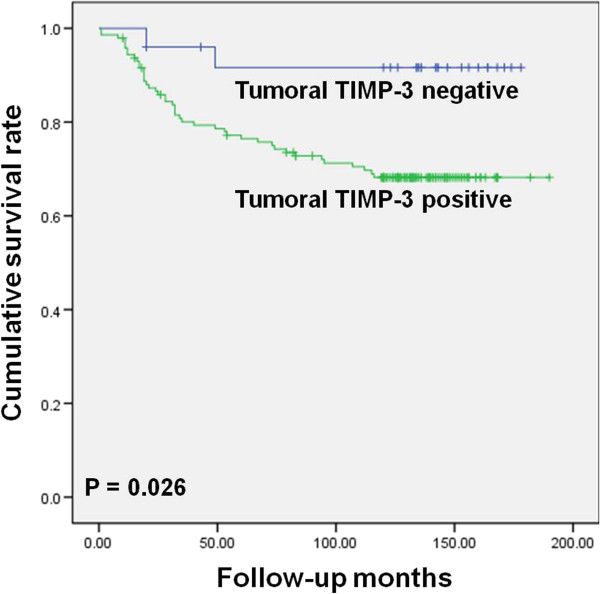
**Kaplan-Meier disease-free survival curves according to tumoral TIMP-3 expression.**

In univariate long-rank analysis, tumor size (*P* = 0.000), status of nodal involvement (*P* = 0.000), tumor stage (*P* = 0.000), and stromal MMP-1 expression (*P* = 0.047) were significantly associated with overall survival (Figure 3). Tumor size (*P* = 0.000), status of nodal involvement (*P* = 0.000), tumor stage (*P* = 0.000), and tumoral TIMP-3 expression (*P* = 0.026) were significantly associated with disease-free survival (Figure 4). All statistically significant variables from the univariate analyses were entered into multivariate Cox regression analysis (Table 3). Multivariate analysis subsequently showed that stage and stromal MMP-1 expression were significantly related to overall survival. Stage was of independent prognostic significance for disease-free survival.

**Table 3:** **Multivariate analysis with Cox’s proportional hazards model for prognostic factors in breast cancer patients**

	Overall survival			Disease-free survival		
	HR	95% CI	***P***-value ^*^	HR	95% CI	***P***-value ^*^
Tumor size (≤2 or >2 cm)	2.873	0.85-9.73	0.090	3.153	0.93-10.71	0.066
Lymph node status (negative or positive)	1.088	0.45-2.62	0.851	1.988	0.83-4.75	0.122
Stage (I, II or III)	4.454	2.46-8.06	0.000	3.577	1.926-6.643	0.000
Stromal MMP-1 expression (negative or positive)	0.528	0.29-0.98	0.042	-	-	-
Tumor TIMP-3 expression (negative or positive)	-	-	-	3.003	0.71-12.63	0.133

* Multivariate analysis was carried out on all variables that were found to be significant in univariate analysis.

HR, hazard rate; CI, confidence interval; -, not significant in univariate analysis.

## Discussion

Microarray profiling of invasive breast carcinomas has identified several distinct molecular subtypes of tumors [4–7]. In accordance with this view, we propose that molecular subtypes are likely to contain distinct MMP/TIMP patterns. In this study, we analyzed the differences in the immunoreactivity of MMPs (MMP-1, 2, 7, 9, 11, 13, and 14) and TIMPs (TIMP-1, 2, and 3) in breast carcinoma representing three subtypes, luminal A, HER2-overexpressing, and basal-like, based on immunohistochemical findings. We demonstrated that tumoral MMP-7, tumoral MMP-9, tumoral MMP-13 and stromal MMP-13 expression were statistically significantly different among the three subtypes.

Gene expression profiling with breast carcinomas has identified five distinct subtypes of the disease: luminal A, luminal B, normal breast-like, HER2-overexpressing, and basal-like [4–7]. Although these molecular subtypes correlate with prognosis and response to therapy, the use of gene expression profiling has been limited by issues such as cost, complexity, and technical expertise. Subsequent studies have proposed novel immunohistochemistry panels to classify breast cancer into five distinct subtypes. These panels use five markers (ER-α, PR, HER2, CK 5/6, and EGFR) to categorize molecular subtypes as luminal A (ER-α + and/or PR+ and HER2-); luminal B (ER-α + and/or PR+ and HER2+); HER2-overexpressing (ER-α- and PR- and HER2+); basal-like (ER-α-, PR-, HER2- and EGFR or CK 5/6+); and unclassified (ER-α-, PR-, HER2-, EGFR-, and CK 5/6-) [8, 9].

Several studies have shown that the basal-like and HER2-overexpressing subtypes have a higher histologic grade than the luminal subtype. In addition, the luminal subtype was shown to have better prognosis than the basal-like and HER2-overexpressing subtypes [21, 22]. Likewise, the present study found that the basal-like and HER2-overexpressing subtypes showed a higher histologic grade than the luminal A subtype. However, our study showed no statistically significant difference among the immunohistochemical-based molecular subtypes for overall and disease-free survival.

Therapy targeting the ER or HER2 oncogene is effective for the luminal and HER2-overexpressing subtypes. However, the basal-like subtype is resistant to targeted therapies such as hormonal therapy or trastuzumab therapy [10]. Hence, studies to identify specific targeted therapies for the basal-like subtype of breast carcinoma have been performed. Many individual markers such as stem cell marker Bmi-1, lysyl oxidase-like 2 (LOXL2), FOXC1, α9β1 integrin, and monocarboxylate transporter 1 were studied to find specific markers for basal-like breast carcinoma [23–27]. Lee et al. [28] found that the basal-like type of breast carcinoma displays a distinct promotor methylation pattern. Thus, we wanted to analyze the expression of MMPs and TIMPs in the three immunohistochemical-based subtypes of breast carcinoma to discover a potential therapeutic target of the basal-like subtype of breast carcinoma.

MMPs and TIMPs play a role in cancer progression including tumor growth, invasion and metastasis [13, 14]. Numerous investigators have reported the significance of MMPs and TIMPs in breast carcinoma. Currently, 28 MMPs and 4 TIMPs are known to exist. Among these, MMP-1, 2, 9, 11, TIMP-1, 2 levels have been largely investigated in breast carcinoma tissues.

A few studies have previously investigated the expression of MMPs and TIMPs in various molecular subtypes of breast carcinoma. McGowan and Duffy [17] investigated the mRNA expression of MMPs in breast cancer by analysis of a published database. Using univariate analysis, they reported that, among 17 different MMPs, MMP-1, 9, 12, 14 and 15 were associated with poor outcome. Of the 5 MMPs, only MMP-14 was determined to be an independent predictor of patient outcome. They also investigated the differences of MMP expression between the basal subgroup and other subgroups (normal-type, luminal A, luminal B and HER-2). They found that mRNA expression of MMP-1, 7, 9, 12 and 15 was significantly elevated in the basal type compared with all the other subtypes combined. González et al. [19] performed an immunohistochemical study of MMP-1, 2, 7, 9, 11, 13, and 14 and TIMP-1, 2, and 3 on cancer specimens from 93 patients with luminal A (n = 48) or basal-like (n = 45) lesions. There were no significant differences in the expression of MMPs or TIMPs in the two phenotypes of tumors.

In the present study, we studied the expression MMPs and TIMPs in the luminal A and basal-like subtypes as well as in the HER2-overexpressing subtype, using a greater number of cases than the previous study by González et al. [19]. Because the crosstalk between cancer- and the surrounding stromal-cells is essential to fine tune the invasivity of cancer cells, we analyzed the differences in the immunoreactivity of MMPs (MMP-1, 2, 7, 9, 11, 13, and 14) and TIMPs (TIMP-1, 2, and 3) in the tumor tissue and in the surrounding stromal tissue of the three major immunohistochemical-based molecular subtypes of breast carcinoma.

Our results demonstrated some significant differences in the tumoral and stromal expression of MMPs and TIMPs depending on the immunohistochemical-based molecular subtype. The expression of tumoral MMP-9 was significantly elevated in the HER2-overexpressing subtype compared with the luminal A subtype and the basal-like subtype. The incidence of stromal MMP-13 expression was significantly higher in the HER2-overexpressing subtype than in the basal-like subtype. In agreement with our results, several studies have also shown that MMP-9 and MMP-13 are correlated with HER overexpression. MMP-9 (gelatinase B) is known to play a role in the invasion and metastasis of cancer through degradation of type IV collagen in the basement membrane and by inducing angiogenesis [29, 30]. High MMP-9 expression was associated with HER2 overexpression [31–33]. MMP-13 (collagenase-3) expression in breast carcinomas was first reported by Freije et al. [34]. MMP-13 may play a key role in the MMP activation cascade [35]. Several studies suggested that MMP-13 might play a critical role in bone metabolism and even induce bone metastasis of breast cancer by activating MMP-9 and other enzymes [36–40]. Zhang et al. [41, 42] reported that tumoral MMP-13 is correlated with HER2 expression.

Matrix metalloproteinase -7 degrades type IV collagen, fibronectin and laminin [16]. Dey et al. [43] found that MMP-7 mRNA level was high in the triple negative breast cancer and this result was associated to the loss of PTEN. Our study showed that tumoral MMP-7 expression was significantly higher in the basal-like subtype compared the luminal A subtype as well as the HER2-overexpressing subtype. We also found that the incidence of tumoral MMP-13 expression was significantly higher in the basal-like subtype than in the HER2-overexpressing subtype.

TIMP-3 may be an important component in inhibiting angiogenesis and stimulating apoptosis [13]. Mylona et al. [44] reported that reduced expression of tumoral TIMP-3 protein was correlated with an aggressive tumor phenotype and shortened disease-free survival in lymph-node-positive patients. Likewise, Span et al. [45] found that high tumor levels of TIMP-3 was associated with longer relapse-free survival in breast cancer patients treated with tamoxifen. Conversely, Vizoso et al. [16] reported that TIMP-3 expression by stromal cells correlated positively with the occurrence of distant metastases. Similarly, del Casar et al. [46] demonstrated that stromal TIMP-3 expression was elevated in primary tumors of patients with distant metastasis, although the result was not statistically significant. Jiang et al. [47] also found that TIMP-3 expression was higher in breast cancer with lymph node metastasis than in those without metastasis. However, the explanation for this discrepancy remains unknown. One possible explanation for these differences can be attributed to the different methods used for the assessment of TIMP3 expression. The present study demonstrated that, in a univariate analysis, TIMP-3 expression was significantly associated with shortened disease-free survival in patients with breast carcinoma. However, in a multivariate analysis, tumoral TIMP-3 expression was not determined as an independent prognostic factor for poor disease-free survival (*P* = 0.133).

Vizoso et al. [16] found that high expression of MMP-1 by fibroblasts was associated to the metastases. Przybylowska [48] et al. described that MMP-1 correlated with the local invasion. Conversely, in the present study, stromal MMP-1 expression was determined as an independent prognostic factor for good overall survival.

In the present study, tumoral MMP-7 and tumoral MMP-13 expression was higher in the basal-like subtype than the luminal A subtype or the HER2-overexpressing subtype. However, we could not find the prognostic significance of MMP-7 and MMP-13 in the basal-like subtype. A caveat with our study is that the specificities of commercial antibodies used were not proven using *in situ* hybridization, which may contribute to differences between studies. Our study is also limited by the small number of cases of the basal-like subtype. Therefore, more studies using a much larger sample size, especially those with the basal-like breast carcinoma, are needed to define the potential prognostic role of MMPs and TIMPs in breast carcinoma. Also, additional studies are needed to determine the mechanisms underlying the differences of MMPs expression in the molecular phenotypes of breast cancer.

## Conclusion

Our research demonstrated some significant differences between MMP and TIMP expression in three immumohistochemical-based molecular subtypes. Tumoral MMP-7 and tumoral MMP-13 expression were significantly higher in the basal-like subtype compared to the luminal A subtype or the HER2-overexpressing subtype. Further studies are required to identify the distinct role of MMPs and TIMPs in the basal-like breast carcinoma.

## Abbreviations

MMP: metalloproteinase
TIMP: tissue inhibitors of metalloproteinase (TIMP)
ER-α: estrogen receptor-α
PR: progesterone receptor
HER2: human epidermal growth factor receptor 2
CK: cytokeratin
EGFR: epidermal growth factor receptor.
